# 

**Published:** 1917-11-10

**Authors:** 


### CONTENTS.

PAGE

**Medicine in the Great War** ... ... ... ... 105

**A Vital Question for Voluntary Hospitals** ... 106

**Hospital and Institutional News** ... ... ... 107

National Relief Fund: Wanted a Member of Parliament—A Portable War Hospital—The Proceeds of "France's Day"—National Service Medical Examination—The South African Ambulance at Cannes—Orthopædic Work in Leeds—The Gay Hospitals of Birmingham—A Ray of Hope for Poor-Law Reformers—Sanatoria and Public Spirit—Record Rain and Record Health—The Technique of the Orderly—Hypnotism and Inflammation—Salary of an Assistant Officer—"Recalled to Life"—The Doncaster Infirmary—The Welsh National Memorial Association—Chaplains' Salaries—The Royal Albert Institution—Hospitals Provided at Short Notice—How the Controversy Ended—Durban as a Military Station—Manual Training in Shell-shock—Tommy's Note-paper and Other Schemes—Real War-babies—Industry and Motherhood—This Week's Drug Market—To Our Readers.

**Health Problems and the State** ... ... ... 113

A Ministry of Health and its Operations.

**The Editor's Letter-Box** ... ... ... ... 114

PAGE

**Two Types of Medical Leader** ... ... ... ... 115

No. 2.—The Humbug.

**Hun Surgeon's Murder** ... ... ... ... 116

**Practical Food of Real Value** ... ... ... 117

II. Why and Where Hospitals Cost More.

**Parliament and the Profession** ... ... ... 118

**Hospital Architecture and Construction** ... 119

Women's Hospital, Dundee.

**Nursing Affairs in Ireland** ... ... ... ... 120

Miss Matheson's Address.

**The Matrons' and Sisters' Department** ... 121

The Nightingale School and St. Thomas's Hospital.

Round the Hospitals.

The R.B.N.A. and its Charter—The College Register—Its Representative Character and Strength—"Ierne" and the College Board for Ireland—What Most Interests Sisters and Nurses—Why Persian Ladies may Enter a Hospital.

**The College of Nursing, Limited...** ... ... 124

**The Royal British Nurses' Association** ... 125

The Story of Recent Happenings.

**Matrons' and Sisters' Appointments** ... ... 128

**The Institutional Worker** ... ... (Suppl.) 1

Patriotic Christmas Cookery.

